# Vitrectomy for diabetic macular edema and the relevance of external limiting membrane

**DOI:** 10.1186/s12886-021-02095-y

**Published:** 2021-09-15

**Authors:** Ivastinovic Domagoj, Haas Anton, Weger Martin, Seidel Gerald, Mayer-Xanthaki Christoph, Lindner Ewald, Guttmann Andreas, Wedrich Andreas

**Affiliations:** Department of Ophthalmology, Medical University Graz, Auenbruggerplatz 4, 8036 Graz, Austria

**Keywords:** Vitrectomy, Diabetic macular edema, Epiretinal membrane, External limiting membrane, Internal limiting membrane

## Abstract

**Purpose:**

To evaluate the relevance of external limiting membrane (ELM) on the visual and morphological results in eyes with diabetic macular edema (DME) that underwent pars plana vitrectomy (PPV) with epiretinal membrane (ERM) and internal limiting membrane (ILM) peeling.

**Methods:**

Medical records of patients with DME who underwent PPV at our unit between January 2017 and December 2019 were reviewed. We assessed preoperative and postoperative best-corrected visual acuity (BCVA), central macular thickness (CMT) using spectral domain OCT (optical coherence tomography). Exclusion criteria were previous PPV; incomplete data; concomitant diseases including retinal vein occlusion, age-related macular degeneration, uveitis; and a follow-up of less than 12 months. The surgeries were performed using 23- or 27-gauge vitrectomy. The ELM was graded depending on its configuration (grade 0 = intact, grade 1 to 3: disruption of varying extent).

**Results:**

Ninety-nine eyes were enrolled. The postoperative follow up averaged 23.7 months. The preoperative and final BCVA averaged 0.71 ± 0.28 and 0.52 ± 0.3 logMAR, respectively (*p* = 0.002). The CMT averaged 515.2 ± 209.1 μm preoperatively and 327 ± 66.1 μm postoperatively (*p* = 0.001). Eyes with intact ELM (*n* = 8) had a significantly better BCVA compared to those with ELM disruption (0.28 ± 0.14 vs. 0.7 ± 0.25 logMAR, *p* = 0.01). The final CMT was similar among the groups (intact ELM: 317 ± 54.6 μm; ELM disruption: 334 ± 75.2, *p* = 0.31).

**Conclusions:**

PPV with ERM and ILM peeling is an effective treatment of DME. Eyes with intact ELM preoperatively had a significantly better final visual outcome. To maximize the benefit for patients with DME we recommend early PPV as long as ELM is intact.

## Introduction

Diabetic macular edema (DME) is a complication of diabetes and represents one of the leading causes of legal blindness. Characteristic features of DME include an abnormal intra- and eventually sub-retinal fluid accumulation in the macula secondary to the blood retinal barrier break-down, pericyte loss and endothelial cell junction breakdown [1]. Currently, DME is predominantly treated with intravitreal anti-VEGF or corticosteroid injections [2–5]. The choice of intravitreal agents as first-line treatment in treatment naive patients with DME depends on several factors including the age, lens status, intraocular pressure and recent cardiovascular events. However, specific structural OCT biomarkers may additionally guide the choice of treatment and monitor the therapeutic response [2]. The effectivity of intravitreal treatment can significantly decrease over time, especially in presence of concomitant vitreoretinal interface pathology including traction, thickened vitreous cortex or epiretinal membrane (ERM) [6, 7]. ERMs in particular occur frequently in eyes with DME and play a significant role in the modulation of DME [8–16]. First, the glial cells of ERMs express various cytokines and growth factors including VEGF and thus contribute to the maintenance of DME [14, 15]. Second, ERMs serve as a mechanical barrier which reduces the permeability of intravitreal anti-VEGF and steroids through the ERM [16].

Pars plana vitrectomy (PPV) combined with ERM peeling is generally an effective treatment in eyes with chronic DME that are refractory to intravitreal injections in terms of visual improvement and decrease of macular thickness [13, 17–23]. However, the postoperative visual outcome varies significantly among eyes depending on the functionality of photoreceptors which is reflected in the configuration of the ellipsoid zone (EZ) and the external limiting membrane (ELM) [23–26]. For example, eyes with intact EZ and ELM commonly show a significantly better postoperative visual outcome than those with disrupted EZ and ELM [23–26]. In order to maximize the benefit for patients with DME and ERM the physicians are thus encouraged to advocate early PPV as long as EZ and ELM are intact. However, before this evidence becomes a decision-making support in the daily routine, its reliability should be tested sufficiently.

The aim of our study is to evaluate whether the ELM configuration had an impact on the long-term postoperative visual and morphological outcome in a routine clinical setting. The focus was set on ELM since its predictive value for visual outcome following PPV in eyes with DME was shown to be slightly superior compared to EZ [23]. The ELM might additionally be more suitable as a preoperative prognostic biomarker since in contrast to EZ the ELM commonly does not restore after PPV [17, 19, 23].

## Methods

This study was approved by the local ethics committee and adhered to the tenets of the Declaration of Helsinki. We retrospectively reviewed medical records of patients with DME who underwent PPV at the Department of Ophthalmology, Medical University Graz between January 2017 and December 2019. The inclusion criteria were a DME refractory to intravitreal agents due to a visible ERM and a central macular thickness (CMT) > 300 μm; complete data including preoperative and postoperative best-corrected visual acuity (BCVA), slit lamp examination, indirect ophthalmoscopy and applanation tonometry, optical coherence tomography (OCT); and a postoperative follow up of at least 12 months. Exclusion criteria were previous PPV; incomplete data; insufficient quality of OCT image; signs of concomitant diseases that might be accompanied with macular edema including retinal vein occlusion, age-related macular degeneration, uveitis; and a follow-up of less than 12 months. The number of intravitreal injections prior and after the surgery were also assessed. The patients’ records were additionally reviewed for demographic data including age and gender, type and duration of diabetes mellitus, HbA_1c_, stage of diabetic retinopathy and Body-Mass-Index calculated as weight/height (in m)^2^.

Spectral domain OCT was conducted with OCT Spectralis version 6.0.9 software (Heidelberg Engineering, Heidelberg, Germany) using volume scanning with 25 sections covering a field of 20 × 20° in the macular region. The device used a bandwidth of 297 nm and a wavelength of 815 nm. Sections were received using the high-speed mode with a resolution of 7 μm axially × 14 μm laterally and a distance of 240 μm between sections. The CMT was automatically calculated with the built-in software. The ELM was assessed foveally in the area 500 μm in either direction from the center of the fovea as previously described [24]. The configuration of ELM was graded from 0 to 3 depending on the extent of ELM disruption, defined as the loss of the line, in the assessed area of 1000 μm. Accordingly, grade 0 was defined as intact ELM, grade 1 as disruption of ELM in up to one third, grade 2 as disruption of ELM in up to two thirds and grade 3 as complete ELM loss. The grading was performed independently by two experts (D.I. and A.G.). The results of their grading corresponded completely.

All surgeries were performed by the same surgeon (D.I.) using the 23-gauge (Oertli OS4, Berneck, Switzerland) or 27-gauge three-port system (DORC, Zuidland, Netherlands). In all eyes PPV was combined with peeling of both, ERM and internal limiting membrane (ILM). The membranes were stained with ILM-Blue® and removed with a 27-gauge extended reach – wide grip microforceps (both produced by DORC). Triamcinolon (Volon® A 10 mg, Mibe GmbH, Brehna, Germany) was used to stain the posterior hyaloid where needed. Phacoemulsification with implantation of a monofocal intraocular lens into the capsular bag was combined with PPV in every phakic patient. At the end of the surgery, all patients received 7 mg betamethasone (Diprophos® 1 ml suspension containing 5 mg betamethason as dipropionate and 2 mg bethamethason as disodium pyrophosphate, Merck Sharp & Dohme GmbH, Vienna, Austria) and 5 mg cefazolin (Kefzol®, Eli Lilly, Vienna, Austria) in the parabulbar space. Postoperatively, the patients were visited on day 1 and dismissed from the hospital depending on the clinical finding. Examinations in the following weeks were performed by the referring ophthalmologist. Each patient was scheduled at our department 1 month after the surgery to perform a complete examination including BCVA measurement, slit lamp examination including indirect ophthalmoscopy and applanation tonometry, and OCT. The intravitreal treatment was continued in case of evident DME. The choice between anti-VEGF and dexamethasone was at physicians’ discretion.

The main outcome measures were BCVA and CMT in μm in dependence of ELM configuration. The BCVA was initially measured in Snellen lines and converted in logMAR to ease the statistical analysis. The descriptive data are presented as the mean ± standard deviation (range). Normal distribution was assessed with Kolmogorov-Smirnov test before every testing. Depending on distribution, intra-group differences were calculated with paired samples t-test or Wilcoxon-test and inter-group differences with independent t-test or Mann Whitney U-test. In case of small sample size, the *p*-value was not calculated. Correlations between various parameters were determined by using Spearman correlation analysis. The statistical analysis was performed using SPSS (IBM, SPSS Statistics 26, New York, USA). The statistics were two-tailed. The threshold for significance was defined as *p* < 0.05.

## Results

**Fig. 1:**
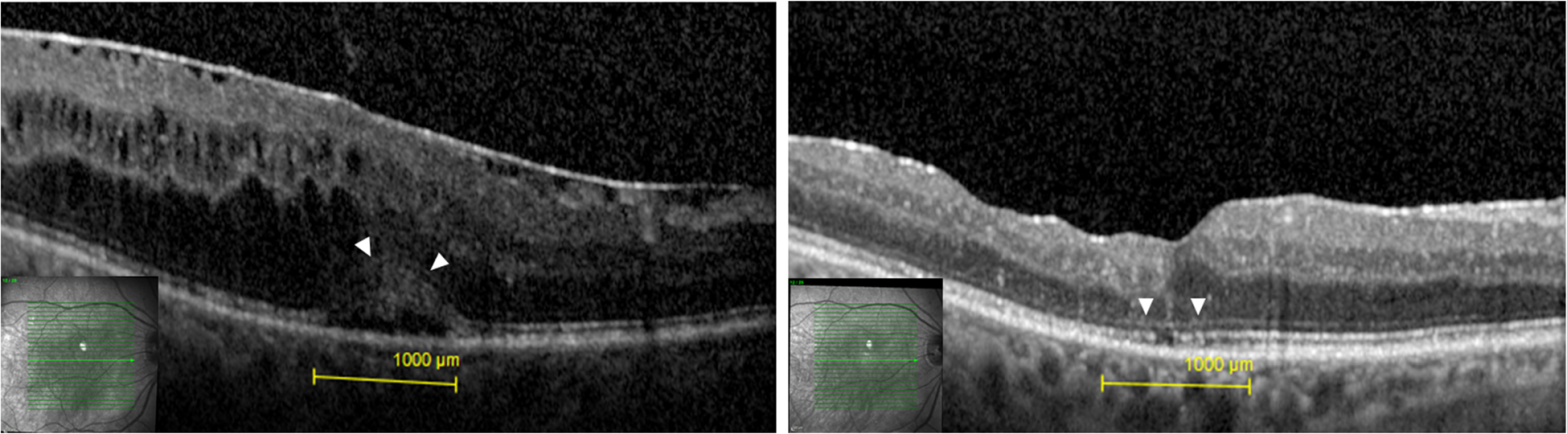
**a** Preoperative OCT with an intact external limiting membrane (ELM) (arrowheads). **b** Postoperative OCT of the same patient 22 months after the surgery. The ELM remained intact (arrowheads). BCVA = best corrected visual acuity, CMT = central macular thickness

**Fig. 2:**
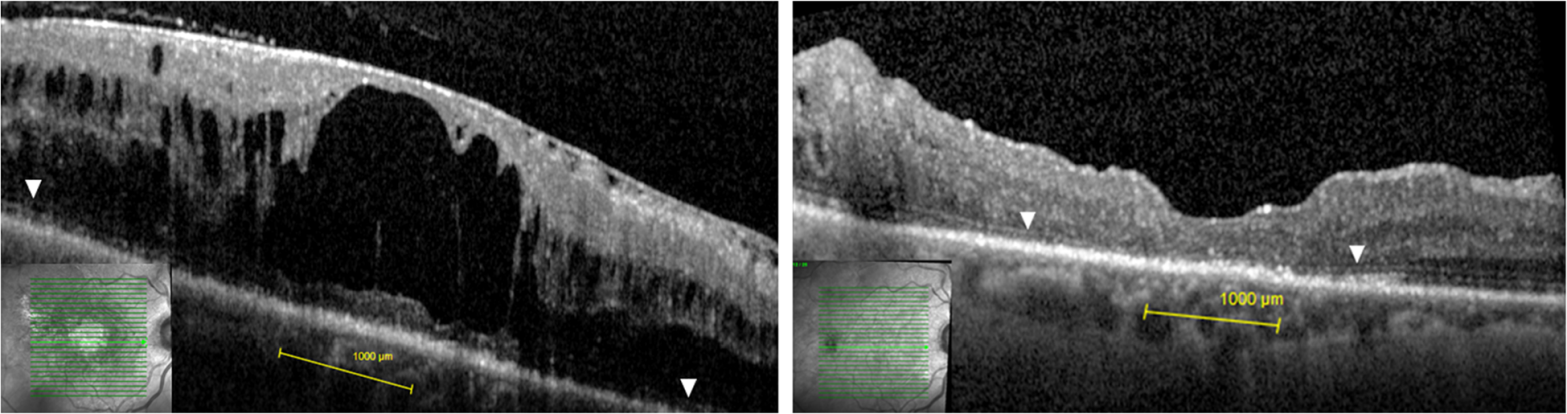
**a**. Preoperative OCT with complete external limiting membrane (ELM) disruption (grade 3) in the fovea. The arrowheads indicate ELM outside the center. **b** Postoperative OCT of the same patient 21 months after the surgery. The ELM remained disrupted in the center. The arrowheads indicate an intact ELM outside the center. BCVA = best corrected visual acuity, CMT = central macular thickness

We enrolled 19 eyes of 17 patients in this study. The mean age of the patients was 69.3 ± 5 years (60–79). Four patients (23.5%) were female and 13 (76.5%) were male. All patients had diabetes mellitus type 2. All eyes were treated with repetitive anti-VEGF intravitreal injections prior to surgery. The average number of preoperative anti-VEGF intravitreal injections was 9.4 ± 6.4 (3–26) over a period of 26.1 ± 22 (3–65) months. Five eyes were additionally treated with 4.0 ± 2.1 (2–7) dexamethasone intravitreal injections (Ozurdex®, Allergan, Ireland) over a period of 28.2 ± 11.7 (14–43) months. In 2 eyes a focal laser was performed previously. The postoperative follow up of averaged 23.7 ± 9.4 (12–44) months. All eyes showed ERM prior to surgery (Figs. 1 and 2). In 8 eyes (42.1%) the ELM was intact (grade 0) and in 11 eyes (57.9%) some degree of ELM disruption was observed (Fig. 1). ELM disruption grade 1 was observed in 4 eyes (21.1%), grade 2 in 3 eyes (15.8%) and grade 3 in 4 eyes (21.1%). Postoperatively, anti-VEGF treatment was continued in 10 eyes and in 2 of these eyes Ozurdex® was additionally applied due to reduced effectivity of anti-VEGF. The average number of postoperative anti-VEGF and Ozurdex® intravitreal injections was 4.7 ± 5.7 (0–16) over 21.6 ± 10.5 (12–44) months and 3.0 ± 2.1 (1–6) over 25 ± 9.1 (13–41) months, respectively. The difference between the preoperative and postoperative number of anti-VEGF injections was statistically significant (*p* = 0.022). The intraocular pressure averaged 14.1 ± 2.6 (10–20) mmHg preoperatively and 14.3 ± 3 (10–21) mmHg at the final visit. We did not observe complications such as endophthalmitis, vitreous hemorrhage or retinal detachment. Table 1 displays the baseline characteristics of enrolled patients depending on the status of ELM.

**Table 1:** Overview of patients’ characteristics

	Intact ELM (*n* = 8)	Disrupted ELM (*n* = 11)	*P* value
Age in years (range)	67 ± 4.2 (60–71)	70.9 ± 5.1 (64–79)	0.11^a^
HbA_1c_ in % (range)	6.9 ± 0.9 (5.8–9)	6.8 ± 0.6 (5.8–7.4)	0.73^a^
BMI in kg/m^2^ (range)	28.9 ± 3.8 (23–33)	28.9 ± 5.1 (21–37)	0.9b
Diabetic retinopathy (DR)			
*Mild*	0	1	
*Moderate*	2	3	
*Severe*	3	2	
*Proliferative*	3	5	
Duration of DME until PPV in months	37.9 ± 25.3 (12–77)	44.9 ± 26.9 (5–94)	0.9b
Duration of follow up in months			
*Preoperative (range)*	30.4 ± 22.1 (6–63)	31.9 ± 21.6 (3–65)	0.88^a^
*Postoperative (range)*	19.9 ± 9.9 (12–41)	26.5 ± 8.4 (17–44)	0.15^a^

^a^independent t-test

^b^Mann Whitney U-test

In general, BCVA averaged 0.71 ± 0.28 (1.1–0.2) logMAR preoperatively and 0.52 ± 0.3 (1–0.1) logMAR at the final visit (*p* = 0.002, Wilcoxon test). In 12 eyes phacoemulsification was combined with PPV. In eyes that underwent phacoemulsification BCVA improved by 1.4 Snellen lines and in eyes that already were pseudophakic by 1.3 lines. The CMT averaged 515.2 ± 209.1 (313–1054) μm preoperatively and 327 ± 66.1 (179–435) μm postoperatively (*p* = 0.001, Wilcoxon test). The preoperative and final measures depending on the ELM configuration are displayed in Table 2.

**Table 2:** Preoperative and postoperative results depending on the ELM status

	Intact ELM (*n* = 8)	Disrupted ELM (*n* = 11)	*P* value
BCVA in logMAR			
*Preoperative*	0.49 ± 0.26 (1–0.2)	0.86 ± 0.16 (1.1–0.7)	0.004^b^
*Postoperative*	0.28 ± 0.14 (0.5–0.1)	0.7 ± 0.25 (1–0.3)	0.01^b^
P value	0.041 ^c^	0.017 ^c^	
CMT in μm			
*Preoperative*	388.1 ± 60.7 (313–486)	607.6 ± 231.9 (364–1054)	0.005^b^
*Postoperative*	317 ± 54.6 (260–429)	334.3 ± 75.2 (179–435)	0.31^b^
P value	0.012 ^c^	0.003 ^c^	

^a^independent t-test

^b^Mann Whitney U-test

^c^Wilcoxon test

The ELM grading in general strongly correlated with the preoperative BCVA (*r* = − 0.719, *p* = 0.001), and final BCVA (− 0.734, *p* < 0.001). A moderate correlation was noted between ELM grading and preoperative CMT (*r* = 0.576, *p* = 0.01). No correlation was observed between ELM grading and the final CMT (*r* = 0.164, *p* = 0.502).

## Discussion

Our study shows that PPV including ERM and ILM peeling generally leads to significant improvement of BCVA and CMT in eyes with nontractional DME with ERM over a mean period of 2 years. The integrity of ELM proved to be a suitable biomarker for visual improvement. Accordingly, eyes with an intact ELM experienced a larger gain in BCVA and had a significantly better final BCVA compared to eyes with ELM disruption (Table 2). In addition, eyes with an intact ELM also had a preoperatively less swelling of the macula compared to those with disrupted ELM. However, no difference was noted in final CMT among eyes with intact or disrupted ELM (Table 2). This finding confirms the evidence that preoperative CMT alone is not predictive for postoperative BCVA improvement [18, 23].

So far, numerous biomarkers have been assessed in respect to their predictive value for postoperative outcome in DME [18, 23–28]. The configuration of ELM and EZ reflect the functionality of photoreceptors and are thus most predictive [23]. ELM is a pseudomembrane formed by adhesions between the inner segments of photoreceptors and Müller cells and EZ represents the density of mitochondria in the inner portions of photoreceptors [29]. In DME, the configuration of ELM proved to slightly better predict the postoperative vision than that of EZ in DME, which could be attributed to the fact that ELM also serves as diffusion barrier between the subretinal space and the inner retina [23, 29]. Consequently, ELM disruption facilitates the migration of proteins, fluid and lipids from the subretinal space into the inner retina and thus contributes to the severity of DME [29]. We are aware that DME is a complex disease and that also other factors including disorganization of retinal inner layers (DRIL) and subretinal fluid (SRF) also contribute to the postoperative outcome [27, 28]. In our study, DRIL was especially observed in eyes with larger extent of ELM disruption (Fig. 1) and eyes with SRF tended to end up with a better BCVA. In advanced DME many morphological retinal changes are present at the same time. The focus of our study, however, was to assess the relevance of ELM alone in eyes that underwent PPV for DME.

PPV was combined with ILM peeling in all eyes. ILM serves as scaffold for ERM and its removal consequently prevents the recurrence of ERM [18, 20, 21]. This aspect is of particular interest in eyes with DME since repetitive intravitreal injections can promote formation of secondary ERM due to upregulation of connective tissue growth factor and fibrosis-related cytokines [6, 30]. In addition, ILM peeling induces some Müller cell injury which consequently promotes a cascade of protective and regenerative reactions including upregulation of the epidermal growth factor receptor and glial fibrillary acidic protein which ultimately results in attenuation of hypoxic damage, reduction of neural cell loss and repair of synapses [31–33]. On the other side, ILM peeling in eyes with DME potentially causes a substantial damage to Müller cell, which can result in macular atrophy, defined as CMT < 220 μm, in approximately one third of eyes [18, 34, 35]. In our study, we observed macular atrophy in 3 eyes (15.8%). However, all affected eyes showed ELM disruption grade 3 and large intraretinal cysts prior to surgery (Fig. 1). Hence, macular atrophy presumably occurred because of advanced DME rather than due to ILM peeling alone.

Our study implicates that PPV should be performed as long as ELM is intact. Therefore, the vitreoretinal interface and the ELM should be carefully observed throughout the treatment with intravitreal injections. In case of ERM formation, PPV with ILM peeling should be performed rather early in order to maximize the benefit for patients. When PPV is used as the last option, the results are commonly disappointing due to compromised microstructure of the macula [17, 23, 25]. Eyes with DME are more likely to develop ERM [8–13]. A recent study even revealed that all eyes with DME show some degree of ERM formation when assessed with electron microscopy and immunohistochemistry, even when no ERM was detectable on OCT. [13] Recently, PPV was even used as first-line option in eyes with treatment naïve DME without evident ERM [18]. This study showed that shorter duration from DME diagnosis to PPV is crucial to achieve a satisfactory visual outcome [18]. In eyes with long lasting DME the visual results were less satisfactory due to the outer retinal damage [18]. Interestingly, in the latter study none of the enrolled eyes (*n* = 120) required intravitreal injections or macula laser after PPV over a period of 2 years [18]. Contrary to these results, we discontinued intravitreal injections in only 5 eyes (26.3%) due to anticipated ineffectiveness based on microstructural macular damage. The number of anti-VEGF injections received after PPV was significantly lower. However, we did not exactly evaluate the impact of postoperative intravitreal injections on the final outcome since this would be beyond the scope of this study. Based on our experience we assume that the majority of eyes would still require intravitreal injections after PPV, however in a lower number than preoperatively.

Early PPV offers additional benefits in eyes with DME. For example, PPV leads to hyperoxygenation of the vitreous cavity and thus increases the oxygen supply to the retina and additionally decreases the level of oxidative stress [36, 37]. This is of particular interest since the oxygen tension in the vitreous of diabetic patients is significantly lower than in nondiabetic controls, even if panretinal laser photocoagulation was performed previously [38]. Moreover, vitrectomized eyes additionally show approximately 2000 times lower viscosity of the vitreous cavity, which facilitates the clearance of VEGF and cytokines from the retina into the vitreous cavity and thus additionally contributes to the resolution of DME [33, 39, 40]. However, it should be considered that hyperoxygenation after PPV can cause oxidative stress and reduce the outflow capacity of the trabecular meshwork and consequently cause ocular hypertension or even glaucoma in up to 6.3% [41]. In addition, retinal detachment occurs after PPV in 4.3% of eyes in diabetic patients [42]. In our study we did not observe any case with ocular hypertension or retinal detachment.

Our study has several limitations including its retrospective design and a low number of eyes. These facts certainly reduce the validity of our study. The limited power of our study additionally compromised the assessment of valid odds ratios for various factors for the BCVA improvement of ≥2 Snellen lines. In this regard a regression analysis, for example, revealed an odds ratio for ELM disruption (grade 1 to 3) of 2.11; however, the 95% confidence interval ranged widely between 0.32 and 13.9 and it was not statistically significant (*p* = 0.44). Despite the limited number of eyes, our study clearly identified the predictive value of ELM in eyes that underwent PPV for DME. In addition, the statistical power was sufficient to show statistical significance in the main parameters including BCVA, CMT and its correlation with ELM grading. Moreover, the study reflects a real-life setting, which is valuable for the clinical routine and the mean follow up of approximately 2 years (median 21 months), is comparably long. However, although PPV with ILM peeling generally showed to be beneficial over the observed period of approximately 2 years, we cannot exclude a development of macula atrophy with consecutive vision decline secondary to the ILM peeling in the following years.

In conclusion, PPV with ILM peeling is beneficial in eyes with DME in functional and anatomical regard. The configuration of ELM proved to be a good predictive biomarker. Therefore, we advocate early PPV in eyes with DME and ERM resistant to intravitreal treatment as long as the ELM is intact.

## Data Availability

The datasets generated and/or analyzed during the current study are not publicly available but are available from the corresponding author on reasonable request.

## Abbreviations

Anti-VEGF: Anti-vascular endothelial growth factor
BCVA: Best-corrected visual acuity
CMT: Central macular thickness
DME: Diabetic macular edema
DRIL: Disorganization of retinal inner layers
ELM: External limiting membrane
ERM: Epiretinal membrane
EZ: Ellipsoid zone
ILM: Internal limiting membrane
OCT: Optical coherence tomography
PPV: Pars plana vitrectomy
SRF: Subretinal fluid
