# Correction to: Vitrectomy for diabetic macular edema and the relevance of external limiting membrane

**DOI:** 10.1186/s12886-021-02118-8

**Published:** 2021-10-25

**Authors:** Domagoj Ivastinovic, Anton Haas, Martin Weger, Gerald Seidel, Christoph Mayer-Xanthaki, Ewald Lindner, Andreas Guttmann, Andreas Wedrich

**Affiliations:** Department of Ophthalmology, Medical University Graz, Auenbruggerplatz 4, 8036 Graz, Austria

**Correction to: BMC Ophthalmol 21, 334 (2021)**

**https://doi.org/10.1186/s12886-021-02095-y**

Following the publication of the original article [1], we were notified that the authors’ first and last names were swapped.

- Originally published names: Ivastinovic Domagoj, Haas Anton, Weger Martin, Seidel Gerald, Mayer-Xanthaki Christoph, Lindner Ewald, Guttmann Andreas and Wedrich Andreas
- Corrected names: Domagoj Ivastinovic, Anton Haas, Martin Weger, Gerald Seidel, Christoph Mayer-Xanthaki, Ewald Lindner, Andreas Guttmann and Andreas Wedrich

The original article has been corrected.

## References

[1] Ivastinovic D (2021). Vitrectomy for diabetic macular edema and the relevance of external limiting membrane. BMC Ophthalmol.

